# Effect of mixotrophic growth on the ultrastructure and fatty acid composition of the diatom *Synedra acus* from Lake Baikal

**DOI:** 10.1186/2241-5793-21-15

**Published:** 2014-08-04

**Authors:** Sergey M Shishlyannikov, Igor V Klimenkov, Yekaterina D Bedoshvili, Ivan S Mikhailov, Alexander G Gorshkov

**Affiliations:** Limnological Institute, Siberian Branch, Russian Academy of Sciences, 3, Ulan-Batorskaya, St, P.O. Box 278, Irkutsk, 664033 Russia

**Keywords:** *Synedra acus*, Lake Baikal, Ultrastructure, Lipid bodies, GC-MS, Fatty acids

## Abstract

**Background:**

Interest in studies concerning the effect of organic carbon sources on the growth of diatoms is largely aimed at subsequent physiological changes occurring in their cells. There are no data on structural changes in the cytoplasm and their relationship with changes in the composition of fatty acids in the course of mixotrophic culturing of freshwater diatoms. To elucidate the role of lipids in the growth of diatom cells in autotrophic and mixotrophic cultures, it is necessary to obtain information on the distribution of fatty acids among intracellular compartments and on possible ultrastructural changes in the cells.

**Results:**

In this study, the results demonstrated that *Synedra acus* cells cultured in the presence of 80 mM glycerol contained lipid bodies of increased size, while cells from cultures supplemented with 40 mM glucose accumulated polysaccharide (chrysolaminarin) granules. An increase in the relative amounts of palmitic and stearic acids was revealed at the exponential growth phase of *S. acus* in the medium with 80 mM glycerol, which was indicative of the accumulation of fatty acids contained in triacylglycerols.

**Conclusions:**

*Synedra acus* subsp. *radians* have an ability to proliferate in the presence of high concentrations of organic substances and their transport into cells is evidence for its high adaptation potential, which, along with other factors, accounts for the dominance of this diatom in the spring-summer plankton of the oligotrophic Lake Baikal.

## Background

Diatoms are autotrophic microalgae that, under certain conditions, can utilize organic carbon sources as nutrients [1–4]. As a rule, these substances are used to store energy as well as to satisfy metabolic needs for cell growth. For example, some species of microalgae assimilate up to 85% of available glucose in the form of reserve polysaccharides [5] whereas other species (e.g. *Prymnesium parvum* and *Dunaliella tertiolecta*) cannot assimilate glucose even though they have the enzyme systems necessary for their metabolism [6]. Glycerol contained in the medium can also be utilized by some microalgae, which provides for an increase in their growth rate [3] and induces certain biochemical [7] and structural changes in the cell, particularly in the morphology of chloroplasts [8].

When nutrient supply is sufficient and environmental conditions are favorable, phytoplankton develops at a high rate, and the greater part of intracellular carbon is in the form of proteins. Under stressful conditions, such as nutrient deficiency (silicon, phosphorus, nitrogen, etc.) [9–11] or insufficient amount of sunlight [12], diatoms begin to store lipids and polysaccharides (in particular, chrysolaminarin) as energy reserve molecules. In the norm, fatty acids in microalgae are utilized mainly for the synthesis of membrane lipids. Under stress, lipid metabolism shifts toward the production of triacylglycerols and enrichment of lipid bodies in the cytoplasm [13].

As shown in previous experiments with diatom cultures, their cells accumulate triacylglycerols as the composition of the medium changes and the age of the culture increases [14, 15], with the resulting increase in the amount of lipids in the cell being accompanied by changes in the composition (ratio) of fatty acids [16–18]. Data on changes in the composition of fatty acids depending on environmental factors provide a basis for the search of new biological indicators characterizing the state of aquatic ecosystems under conditions of climate-dependent changes in the temperature of water bodies and their increasing trophicity resulting from the impact of human activities. Moreover, analysis of the effect of stress factors on the development of diatoms may provide the possibility of optimizing biotechnology of their culturing in order to obtain cell biomass with the desirable composition of low-molecular-weight compounds, fatty acids in particular.

Diatoms, green and red algae are natural producers of value-added bioactive compounds, such as PolyUnsaturated Fatty Acids (PUFAs). Some essential PUFAs including EPA (eicosapentaenoic acid, ω-3, C20:5), DHA (docosahexaenoic acid, DHA, ω-3, C22:6), and AA (arachidonic acid, ω-3, C20:4) have critical physiological functions, e.g., preventing high cholesterol, myocardial infarction and improving high blood pressure, etc. Therefore, PUFAs have attracted a great attention due to their beneficial effects on human health [19]. Microalgae have potential for numerous commercial applications, among them the production of PUFAs [20]. Diatoms normally contain significant amount of EPA and lower levels of DHA [21–23]. Red algae also synthesize large amounts of EPA accompanied with AA [24]. EPA and DHA are typically absent in green algae [25] but these algae contain, in generally, primarily C16 and C18 PUFAs [26, 27].

Interest in studies on the effect of organic carbon sources on the growth of diatoms is largely aimed at subsequent physiological changes occurring in their cells. A fairly detailed analysis has been made of various factors having an effect on the ultrastructure of marine and freshwater diatoms cultured under autotrophic, mixotrophic, and heterotrophic conditions [8, 28–32]. On the other hand, there are no data on structural changes in the cytoplasm and their relationship with changes in the composition of fatty acids in the course of mixotrophic culturing of freshwater diatoms. To elucidate the role of lipids in the growth of diatom cells in autotrophic and mixotrophic cultures (supplemented with organic carbon sources), it is necessary to obtain information on the distribution of fatty acids among intracellular compartments and on possible ultrastructural changes in the cells.

*Synedra acus* subsp. *radians* (Kütz.) Skabitsch, one of dominant species in the phytoplankton of oligotrophic Lake Baikal, has been the object of intensive research on morphogenesis of frustules [33] as well as of molecular [34–36], cytological [37], and genomic studies [38]. As shown previously, this diatom is a potential source of PUFSa, mainly of EPA [39].

The purpose of this study was to analyze changes in the morphology and fatty acid composition of *S. acus* cells cultured in the presence of glucose and glycerol as organic carbon sources.

## Results

**Figure 1:**
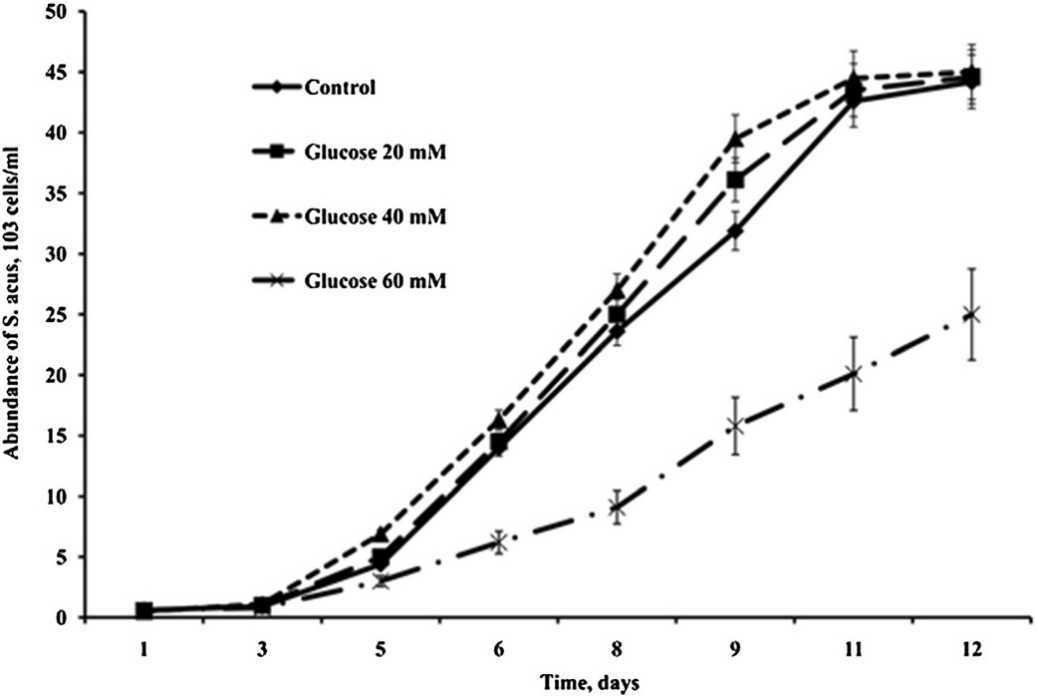
**Dynamics of** ***S. acus*** **abundance in autotrophic and mixotrophic cultures with different concentrations of glucose.**

**Figure 2:**
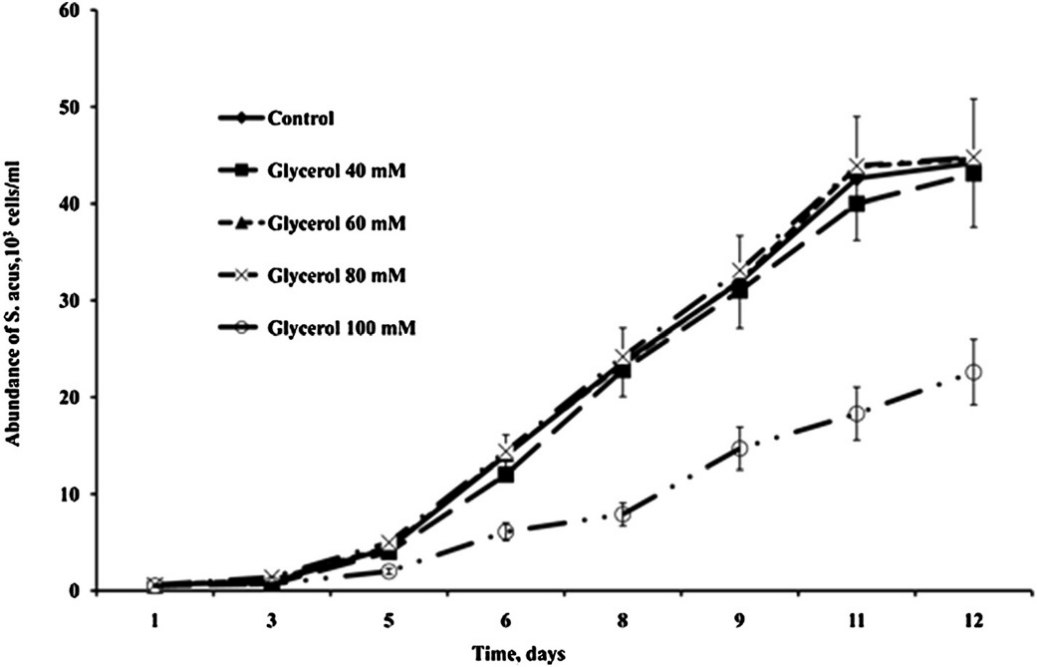
**Dynamics of** ***S. acus*** **abundance in autotrophic and mixotrophic cultures with different concentrations of glycerol.**

In preliminary experiments, the maximum concentrations of organic carbon sources in the medium that did not result in a reduced rate of cell divisions or gradual cell death in *S. acus* culture were estimated at 40 mM for glucose and 80 mM for glycerol (Figures 1 and 2). Therefore, it is these concentrations that were used in subsequent mixotrophic cultures.

**Figure 3:**
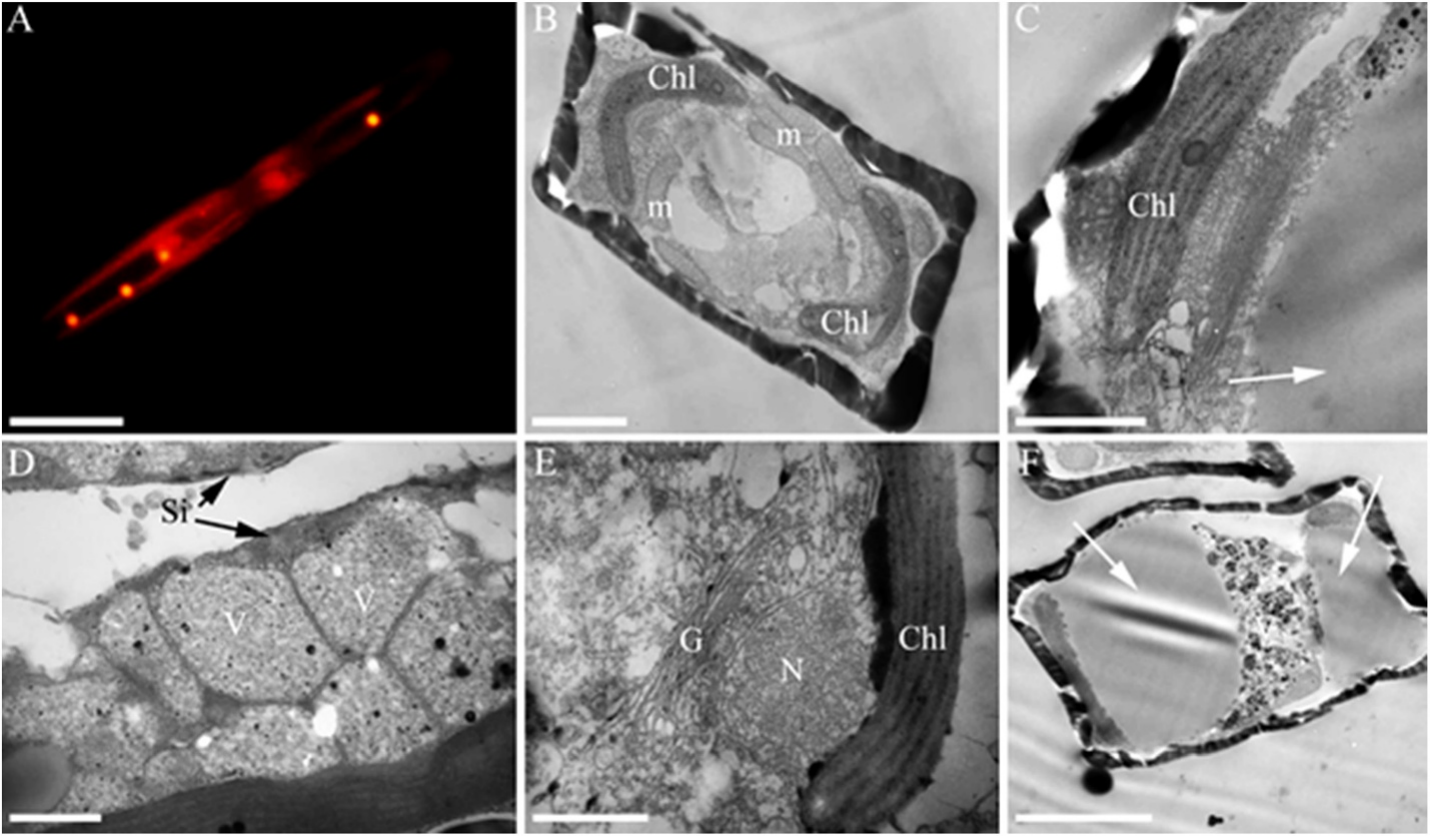
**Fluorescence and transmission electron micrographs of Synedra acus cells.** Yellow fluorescence of lipid bodies stained Nile Red dye and the red autofluorescence of chloroplasts **(A)**, ultrastructural characteristics Synedra acus cells in autotrophic culture **(B, E)** and in mixotrophic cultures with glycerol **(C, F)** and glucose **(D)**. Designations: Chl -chloroplast; V-vacuole with chrysolaminaran granules, m - mitochondria. N- nucleus. White arrows indicate lipid bodies. Scale bars: **(A)** - 10 μm, **(C, D, E)** - 500 nm, **(B,F)** - 2 μm.

According to the results of TEM, *S. acus* cells in autotrophic culture (Figure 3B) formed vacuoles (up to 1.0 μm) with homogeneous electron-dense contents, which could be located either at the periphery or in the center of the cell (Figure 3E). These vacuoles in preparations stained with Nile red were bright orange (Figure 3A), which was evidence for the lipid nature of their contents [40]. Such intracellular lipid bodies were also found in cells cultured under mixotrophic conditions (with glycerol or glucose). It should be noted, however, that the size of lipid bodies in cells from cultures with glycerol was greater than in cells from either autotrophic (control) or glucose-containing cultures, reaching 2.5 μm (Figure 3C and 3F). Such glycerol-dependent accumulation of lipids in the cytoplasm of diatoms was observed already at the phase of their exponential growth. Further enlargement of lipid inclusions at the stationary growth phase resulted in a increase in the overall cell volume, which was usually accompanied by deformation of the cell wall in the girdle zone (Figure 3F). At the stage of frustule formation, cells cultured in the presence of glucose contained a different kind of vacuoles (0.5-0.7 μm), which had heterogeneous granular and small-grained contents (10–20 nm, Figure 3D), and also included more electron-dense granules of a larger size (20–40 nm, Figure 3D). Such structures were not revealed in the cells cultured in autotrophic conditions and with glycerol. The characteristic structure of these organelles [41] could be regarded as evidence for the accumulation of polysaccharides (in particular, chrysolaminarin) in the cells.

Analysis of *S. acus* cell lipids was performed by means of their acid hydrolysis followed by gas chromatography of the extracted fraction of fatty acid methyl esters. It was found that the qualitative composition of fatty acids remained basically unchanged in the course of culture growth (Table 1). Thus, the group of dominant fatty acids at the exponential growth phase included myristic (C14:0), palmitic (C16:0), palmitoleic (C16:1), hexadecatrienic (C16:3) stearic (C18:0), and eicosapentaenoic (C20:5) acids, and that at the stationary growth phase, myristic, palmitic, palmitoleic, and eicosapentaenoic acids. Such composition of fatty acids is typical for hydrolysates of diatom lipids [21, 22].

**Table 1:** **Fatty acid composition (% of total fatty acid) in** ***S. acus*** **biomass from different types of cultures (n = 3)**

Fatty acid (FA)	Autotrophic culture		Mixotrophic culture (40 mM glucose)		Mixotrophic culture (80 mM glycerol)	
	Exponential phase	Stationary phase	Exponential phase	Stationary phase	Exponential phase	Stationary phase
С14:0	12.0	12.9	13.6	17.9	9.8	10.7
С16:0	16.4	10.8	11.4	11.3	24.7	13.4
С18:0	16.9	3.1	10.3	3.7	27.1	4.6
С16:1	10.6	44.2	13.9	44.6	10.8	41.2
С18:1	0.0	0.9	0.0	1.4	0.0	0.7
С22:1	0.0	0.0	1.1	0.0	0.0	0.0
С16:2	5.3	2.7	6.2	1.9	3.8	3.5
С16:3	17.2	5.1	18.6	2.3	9.9	7.0
С18:3	0.0	2.3	0.0	2.4	0.0	1.8
С20:5	19.0	16.8	21.1	13.2	11.3	16.0
С22:6	2.6	1.2	3.8	1.3	2.6	1.1
Σ Saturated fatty acid (SFA)	45.3	26.8	35.3	32.9	61.6	28.7
Σ Monounsaturated fatty acid (MUFA)	10.6	45.1	15.0	46.0	10.8	41.9
Σ Polyunsaturated fatty acid (PUFA)	44.1	28.1	49.7	21.1	27.6	29.4
ΣSFA/Σ(MUFA + PUFA) ratio	0.8	0.3	0.5	0.5	1.6	0.4

As follows from Table 1, ΣSFA/Σ(MUFA + PUFA) ratios in cell lipids markedly differed between cells cultured under photoautotrophic and mixotrophic conditions at the exponential phase. For example, palmitic and stearic acids dominated in the fatty acid spectrum of *S. acus* cells cultured with 80 mM glycerol, indicating that these acids, contained in triacylglycerols, were accumulated in the lipid bodies.

Judging from the ΣSFA/Σ(MUFA + PUFA) ratios of fatty acids in lipid hydrolysates, the relative amount of saturated fatty acids in *S. acus* cells decreased at the stationary growth phase in autotrophic cultures and mixotrophic cultures with 80 mM glycerol but remained unchanged in cultures with 40 mM glucose (Table 1). The contents of monounsaturated fatty acids increased threefold at the stationary growth phase in both autotrophic and mixotrophic cultures with glucose or glycerol. The amount of PUFAs decreased in autotrophic cultures and mixotrophic cultures with 40 mM glucose but changed only slightly in cultures with 80 mM glycerol.

## Discussion

The data presented above show that the fatty acid profile of lipids from diatom cells is subjected to changes, which apparently result from exposure to stress factors. One of such factors may be the addition of organic carbon sources (glucose or glycerol) to the culture medium.

At the stationary growth phase, the content of palmitoleic acid in *S. acus* culture has proved to increase, by more than 40% relative to the total amount of fatty acids (Table 1). This fatty acid is most prevalent in the composition of diatom lipids [21, 42, 43]. Fatty acid biosynthesis in all plastid-containing organisms is catalyzed by type II fatty acyl synthase [44]. Armbrust *et al*. described complete cycles of the biosynthesis of polyunsaturated fatty acids in the marine diatom *Thalassiosira pseudonana* [45]. Fatty acids synthesized in plastids are exported to the cytoplasm to be incorporated into intracellular lipids. It is known that diatom cells in cultures at the stationary growth phase experience a deficit of nutrients and begin to accumulate storage compounds such as lipids, inorganic ions and polysaccharides [11, 46]. When the medium is supplemented with a certain source of carbon, diatoms can build up more biomass due to intensification of lipid biosynthesis [3, 47]. Thus, the increase in the relative content of palmitoleic acid in *S. acus* cells may be regarded as evidence for its accumulation as a component of reserve triacylglycerols stored in lipid bodies. Palmitic, palmitoleic and stearic acids are known to prevail in reserve triacylglycerols accumulated by photosynthesizing cells [18, 48]. In our experiments, the relative contents of stearic, palmitic, acids in the biomass of *S. acus* diatoms at the exponential growth phase were found to be higher in cultures with glycerol than in cultures with glucose or in the control (Table 1), which could be indicative of an increasing accumulation of lipids in *S. acus* cells.

In our experiments, the dominant PUFA was EPA at exponential growth phase (Table 1). The dietary uptake of EPA and DHA is very important in human health. High EPA productivity is typical for many diatoms, for example, *Phaeodactylum tricornutum* [23] *Navicula saprophila* and *Nitzschia* sp. [2]. Therefore, *S. acus* can be a preferable diatom that can be used as producer for “ω-3 polyunsaturated fatty acids” (PUFAs) and the putative candidate for genetic engineering.

These biochemical data are supported by the results of microscopic analysis. TEM preparations of *S. acus* cells from cultures with glycerol at the exponential growth phase, when the cells experience increasing deficit in silica, clearly show that the size of lipid bodies in the cytoplasm increases, compared to the control, and that this increase continues at the stationary growth phase, resulting in the expansion of cell volume in the girdle zone (Figure 3F). This is evidence for the existence of a mechanism for transmembrane transport of glycerol into *S. acus* cells and its involvement in cell metabolism. It is known that glycerol not only has a structural function as a component of cell membrane lipids but also plays a role in energy metabolism (in the form of glycerol-3-phosphate) and regulation of glycerol transport and lipid metabolism, and transmembrane transport of small charged molecules [49].

*Synedra acus* cells cultured in the presence of glucose proved to form polysaccharide granules in the cytoplasm (Figure 3D), which were absent in cells from cultures with glycerol and control cultures. In previous microscopic and biochemical studies, the main polysaccharide stored in the vacuoles of marine and freshwater diatoms was identified as β-1,3-glucan, or chrysolaminarin [41, 50, 51]. This polysaccharide is metabolized during the dark period and thereby serves as an energy substrate for cell metabolism. As shown recently, the genome of the marine diatom *Thalassiosira pseudonana* contains genes coding for еndo- and exo-active β-1,3-glucanases involved in intracellular chrysolaminarin hydrolysis [45]. These data and the results of our experiments indicate that glucose molecules entering the cytoplasm of *S. acus* cells become involved in cell metabolism.

## Conclusions

The results of this study do not provide unequivocal answers to the questions as to whether or how the processes of transport of silica and organic molecules through the diatom cell wall are coupled and to what extent they depend on each other. However, it is clear that *S. acus* cells under conditions of deficit in silica show the intake of molecules providing a source of energy (glucose or glycerol) from the ambient medium. The ability of this freshwater diatom to proliferate in the presence of high concentrations of organic substances and their transport into cells is evidence for its high adaptation potential, which, along with other factors, accounts for the dominance of *Synedra acus* subsp. *radians* in the spring-summer phytoplankton of oligotrophic Lake Baikal.

## Methods

### Diatom culture

The initial axenic *S. acus* culture maintained in the laboratory was isolated from a natural population sampled in Listvennichnyi Bay, Lake Baikal [52]. In this study, *S. acus* cells were cultured in 5-ml tubes and 1-l Erlenmeyer flasks with DM medium [53] at 18°C and natural photoperiod. Test cultures were supplemented with different concentrations of glucose (10, 20, 40, or 60 mM) or glycerol (10, 40, 60, 80, or 100 mM). Autotrophic cultures in DM medium without additives served as control. Cell counts in the cultures were taken daily during 12 days. All experiments were performed in triplicate.

### Light microscopy

Samples from the cultures were examined under an Axiostar Plus microscope (Carl Zeiss, Germany) at 1000× magnification. Microscopic images were made using an AxioCam I Cc1 digital camera with the AxioVision 4.7 program (Carl Zeiss, Germany).

### Fluorescence microscopy

Samples for fluorescence microscopy were taken at the exponential and stationary phases of culture growth (1.5-2.0 × 10^4^ and 3.5-4.0 × 10^4^ cells ml^-1^, respectively). They were supplemented with 10 μl of a 2 mg ml^-1^ stock solution of Nile red stain (Sigma, USA) in acetone, incubated for 5 min, transferred onto a glass slide under a cover slip, and examined under an Axiovert 200 inverted microscope (Carl Zeiss, Germany) with an Osram HBO 50 W/AC mercury lamp. Microscopic images were made using a Penguin 600CL digital camera (Pixera Corp., USA) with the AxioSet program (Carl Zeiss, Germany).

### Transmission electron microscopy (TEM)

Samples for TEM were taken at the exponential and stationary phases of culture growth (1.5-2.0 × 10^4^ and 3.5-4.0 × 10^4^ cells ml^-1^, respectively). They were concentrated on a polycarbonate membrane filter with a pore size of 5 μm (Millipore, Ireland) placed in a Sartorius glass-vacuum holder, 25 mm, 30 ml (Germany) and fixed with glutaraldehyde (Sigma-Aldrich, USA) for 30 min and then with OsO_4_ (Merck, Germany) for 10 min (final concentrations 2.5% and 1%, respectively). Thereafter, the cells were washed in three portions of DM medium, embedded in 1% agar (Helicon, Russia), and dehydrated in an ascending alcohol series (10, 30, 50, 70, 80, and 96%) followed by absolute alcohol and acetone dehydrated with calcined copper sulfate (3 min each). Dehydrated samples were impregnated with mixtures of Araldite 502 epoxy resin (SPI, USA) and acetone (1:2, 1:1, and 2:1, 6 hrs each) and with pure Araldite (12 hrs), transferred to a new portion of Araldite supplemented with DMP-30 accelerator (SPI; 250 μl per 20 ml of the resin), and polymerized in a thermostat at 60°C for 3 days. Ultrathin sections were made in an Ultracut R (Leica, Austria) with an ULTRA 35 diamond knife (Diatom, Switzerland), placed onto palladium grids, and contrasted with lead nitrate as described [54]. TEM analysis was performed using a Leo 906 E microscope (Carl Zeiss, Germany) at an acceleration voltage of 80 kV. Microscopic images were made with a MegaView II camera (Carl Zeiss, Germany) and processed using the MegaVision program.

### Isolation of lipids

*Synedra acus* cells from the culture were collected on a polycarbonate filter as described above, washed with sterile DM medium, transferred to 5-ml plastic tubes with screw caps, and treated with a 2:1 methanol-chloroform mixture (2 ml per 50 mg of cells) at 5°C for 12 hrs. For phase separation, the mixture was additionally supplemented with 1 ml of chloroform and 1 ml of water and incubated overnight at 5°С. The chloroform phase was then collected, dried over anhydrous sodium sulfate, and the solvent was evaporated under argon.

### Preparation of fatty acid methyl esters

Total lipids extracted from diatom cells (10 mg) were suspended in 0.2 ml of 2% H_2_SO_4_ solution in methanol and incubated at 75°C for 1 hr in a sealed tube. The mixture was then cooled, supplemented with several drops of water, and treated with three 0.2-ml portions of n-hexane to extract fatty acid methyl esters. The extracts were pooled and evaporated under argon. Samples for subsequent analysis by means of Gas Chromatography-Mass Spectrometry (GC-MS) were dissolved in 300 μl of n-hexane.

### GC-MS analysis of fatty acid methyl esters

Analysis was performed in an Agilent GC 6890 gas chromatography system with a DB-5MS capillary column (30 m × 0.25 mm, 250 μm film thickness) coupled to an MSD 5973 mass spectrometer (Agilent Technologies, USA), under the following conditions: column heating from 160 to 300°C at 2°C/min, with the temperature maintained at 300°C for 20 min; injector temperature 290°C, quadruple temperature 150°C; injected sample volume 2 μl. Peak detection was performed in the full scan mode in a mass range of 50 to 500 atomic mass units, at a scanning rate of 0.1 s and an ionization energy of 70 eV.
